# Miserable malalignment syndrome associated knee pain: a case for infra-tubercle tibial de-rotation osteotomy using an external fixator

**DOI:** 10.1186/s13018-023-04252-z

**Published:** 2023-10-11

**Authors:** Ahmed A. Elsheikh, George W. V. Cross, Jonathan Wright, William David Goodier, Peter Calder

**Affiliations:** 1 Department of Orthopaedic Surgery, Faculty of Medicine, Benha University, Benha, 13511 Egypt; 2 Limb Reconstruction Unit, Royal National Orthopaedic Hospital, Brockley Hill, Middlesex, UK

**Keywords:** Miserable malalignment syndrome, Tibia de-rotation, External fixator, Anterior knee pain, Femoral anteversion, Tibia torsion

## Abstract

**Introduction:**

Miserable malalignment syndrome is a complex torsional lower limb deformity with limited consensus on surgical treatment. We present the outcome of de-rotation of the tibia alone using an external fixator.

**Methods:**

Fifteen patients (22 segments) were operated on between 2012 and 2020; 13 presented with anterior knee pain, and two presented with out-toeing. Gait analysis was done in nine patients, and CT scan rotational profile, including tibial tubercle–trochlear groove distance, femoral version, and tibial torsion, were calculated. Kujala knee pain score and visual analogue pain score (VAS) were recorded. All underwent infra-tubercular osteotomy of the tibia and midshaft osteotmy of the fibula and application of a hexapod circular frame to gradually internally rotate the tibia until the foot aligned with the patella.

**Results:**

There was no preoperative clinical or radiographic evidence for patellar instability, femoral anteversion 30° (21°–54°), and external tibial torsion 50° (37–70). The mean age at surgery was 21 years (12–37) with a mean follow-up of 20 months (9–83). All osteotomies healed, and the frames were removed at a mean of 111 days (80–168). The mean VAS score improved from 8(5–9) to 1(0–4) postoperatively (*P* < 0.001). The mean Kujala knee pain score increased from 53 (30–75) to 92 (54–100) postoperatively (*P* < 0.001). The mean preoperative foot progression angle (FPA) was 37° (20°–50°), with 13 postoperatively walking with neutral FPA. One patient walked with symmetrical + 10° and the other with − 5° FPA. All patients reported relief of knee pain and were satisfied with the alignment.

**Conclusion:**

Gradual correction of severe external tibia torsion with a hexapod external fixator and an infra-tubercle tibial osteotomy could provide an optimum method to eliminate knee pain and improve limb alignment in miserable malalignment syndrome.

## Introduction

Whereas anterior knee pain remains a common presenting complaint in children, adolescents, and young adult patients, the recognition of lower limb torsional malalignment is often overlooked. It remains an uncommon aetiology compared to conditions such as Osgood–Schlatter disease, Sinding–Larsen–Johansson disease, chondromalacia patellae, and patella instability associated with extensor mechanism malalignment [1, 2]. The term miserable malalignment syndrome (MMS) relates to combined excessive proximal femoral anteversion (PFA) and external tibial torsion (ETT) [3–8]. The rotation effect has been associated with knee pain and symptoms due to increased patellofemoral contact pressures [9–11].

An understanding of what is “normal” lower limb rotation has been documented, both clinically [12] and following radiological assessment [13–16]. Furthermore, indications for the treatment of torsional pathology are suggested, including PFA greater than 50°, hip internal rotation (IR) greater than 85°, or external rotation (ER) less than 10°, and ETT greater than 30° [17]. A question remaining includes which surgical correction method to undertake? A combination of femoral and tibial de-rotational osteotomies has been recommended by some authors [4–6]. Others performed proximal tibial supra-tubercle osteotomy, with realignment of the tibial tubercle focusing on diagnosing patellofemoral instability [2, 18–20]. Distal tibial osteotomy has also been recommended lowering the risk of traction injury to the peroneal nerve [7].

Understanding the patient’s rotational profile and gait preference should identify the PFA, ETT, and relationship of the quadriceps–patella–tibial tubercle alignment (Q angle) [2, 14, 21]. Our hypothesis states that the patient prefers to walk with a neutral foot progression angle and that the severe ETT is compensated by the hip held in internal rotation with a resultant squinting patella. It is accepted that external rotation of the hip will be limited by PFA, but when at least 10° of external hip rotation is present, patients are able to walk with the patella facing forwards and a resultant marked external foot progression with reduced knee pain. We believe, therefore, that the main driving force of knee pain in miserable malalignment is from the compromised inwards-facing knee position, enabling the foot to point forwards. This study presents the outcome of patients with confirmed miserable malalignment treated by an isolated infra-tubercle proximal tibial osteotomy, stabilised with a hexapod external fixation, and the deformity corrected gradually by distraction osteogenesis.

## Patients and methods

A retrospective review of the limb reconstruction database identified fifteen patients (22 tibias) diagnosed with MMS. Thirteen patients presented with debilitating anterior knee pain, and two presented with out-toeing underwent treatment between 2012 and 2020. The study proposal was approved by the research and innovation committee under the registration number: SE21.35.

The patient evaluation included a full clinical examination, which comprised an assessment of the foot progression angle during “normal” gait (the most comfortable gait that the patient used to walk with), and when patients walked with the patella facing forwards (they were asked to focus and keep their patella forward during their walk). The rotational profile of the lower limb was determined in the prone position as described by Staheli [12], including hip rotation in extension and foot–thigh angle.

The radiological assessment included a long-leg standing radiograph with the patella pointing forwards to determine concomitant coronal angular deformity, false-profile lateral of the hips, and lateral views of the tibia to confirm no sagittal deformity. Computed tomography (CT) of the hips, knees, and ankles was undertaken to calculate the femoral neck version of the femoral condyle axis, and tibial torsion in relation of the joint axis to the intermalleolar axis [13–16]. The tibial tuberosity to trochlear groove distance (TT-TG) was also measured using the CT scan to evaluate patellar translation; 12 mm or less was normal, greater than 16 mm was indicative of malalignment, and 20 mm was accepted as the pathological threshold [14].

The Kujala knee pain score [22] and the visual analogue score (VAS) were recorded. Gait analysis was undertaken in nine patients.

### Surgical technique

A general anaesthetic with prophylactic antibiotics on induction was administered. The patient was placed supine; a tourniquet was not used. A mid-diaphyseal fibula osteotomy was performed with an oscillating saw cooled with saline, and the osteotomy was completed with an Ilizarov osteotome. The senior author (PC), an experienced limb reconstruction surgeon, performed all procedures.

A proximal reference wire was placed parallel to the tibial joint surface, 1–1.5 cm from the articular surface. A two-thirds Taylor spatial frame (TSF, Smith and Nephew, Memphis, TN, USA) was fixed to the wire, with adequate anterior space. The ring was orientated perpendicular to the long axis of the tibia, and further fixation with two crossing wires was applied. A complete ring was placed over the mid-diaphysis of the tibia using fast-fix struts. The ring was externally rotated to align with the second ray of the foot, mimicking the ETT. The fast-fix struts were secured, and the ring was fixed to the tibia with a lateral to medial tibial face wire. Fixation was completed with one proximal and one or two distal anteromedial half-pins fixed to the distal ring and a third proximal anteromedial half-pin fixed to the proximal ring. A small 1-cm incision was made over the proximal tibia just below the tibial tubercle. The periosteum was elevated, the tibia pre-drilled, and the osteotomy was completed with Ilizarov osteotomes.

### Postoperative rehabilitation

Following a latency period of 6 days, the osteotomy was initially distracted by 1 mm a day for 1 week, followed by correction of the ETT over 2 weeks. The patient was involved in determining the final correction; with encouragement to walk full weight-bearing, they assessed the alignment of the foot in relation to the patella. They walk with the patella facing forwards, and the correction produces a neutral foot progression angle, with the aim of the patella and foot in alignment. Long-leg alignment views ensured that there was no angular deformity. Once the correction had been accepted, radiographs were repeated at 4 weekly intervals until full consolidation, and the external fixator was removed, either in the outpatient clinic using nitrous oxide or under general anaesthesia. A Sarmiento weight-bearing cast was applied following frame removal for approximately 4 weeks. Kujala knee scores and visual analogue scores were recorded at the last follow-up appointment. Complications during the treatment or following frame removal were recorded. Any further surgical intervention was described in patients following frame removal.

Data management and statistical analysis were undertaken using SPSS version 28 (IBM, Armonk, New York, USA). Quantitative data were summarised as means and ranges. Categorical data were summarised as numbers and percentages. The preoperative versus postoperative values were compared using Wilcoxon signed-ranks test. *P* values less than 0.05 were considered significant.

## Results

The study group consisted of thirteen females and two males, with a mean age of 21 years (range 12–37). Eight patients were skeletally mature adults; the remaining seven were under eighteen. Seven patients underwent bilateral tibial osteotomies, and eight patients underwent unilateral correction, with all but one undertaken on the right side, making a total of twenty-two segments for analysis.

Thirteen patients presented with anterior knee pain, they stood and walked with a neutral foot progression, with a concomitant internal squinting of the patella. They could stand and walk with their patellae pointing forwards which uncovered the ETT. Two patients did not report knee pain but presented with out-toeing which was cosmetically disturbing, and they sought medical advice to correct it. The mean external foot progression angle with the patella facing forward was 37° (range 20°–50°). Clinical examination demonstrated a mean hip IR in the extension of 51° (range 40°–80°) and mean ER of 28° (range 10°–60°). The mean foot–thigh angle was measured at + 40° (range 20°–60°), where positive angle (+) refers to external rotation, and negative angle (−) refers to internal rotation.

The mean femoral anteversion on CT was 30° (range 21°–54°), and the mean ETT was 50° (range 37°–70°). The mean TT-TG measured 10.8 mm (range 5–21 mm, 14 patients were within normal limits, and only one reached the threshold value of 21 mm). The gait analysis confirmed the internal rotation of the femoral segment and the external rotation of the shank.

**Fig. 1:**
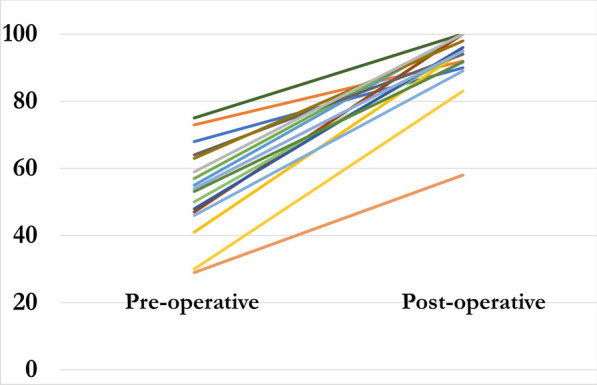
Kujala knee scores (preoperative compared with postoperative)

**Fig. 2:**
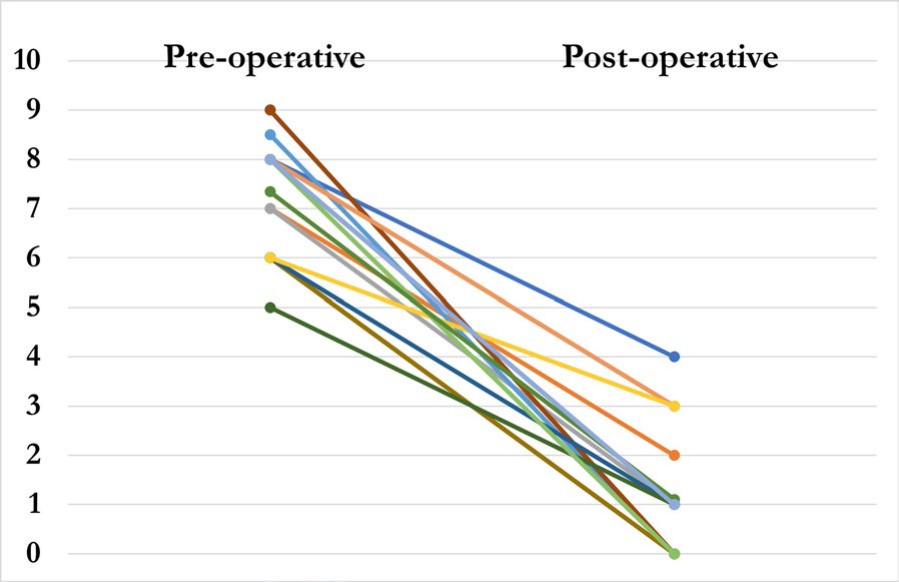
Visual analogue score (preoperative compared to postoperative)

**Fig. 3:**
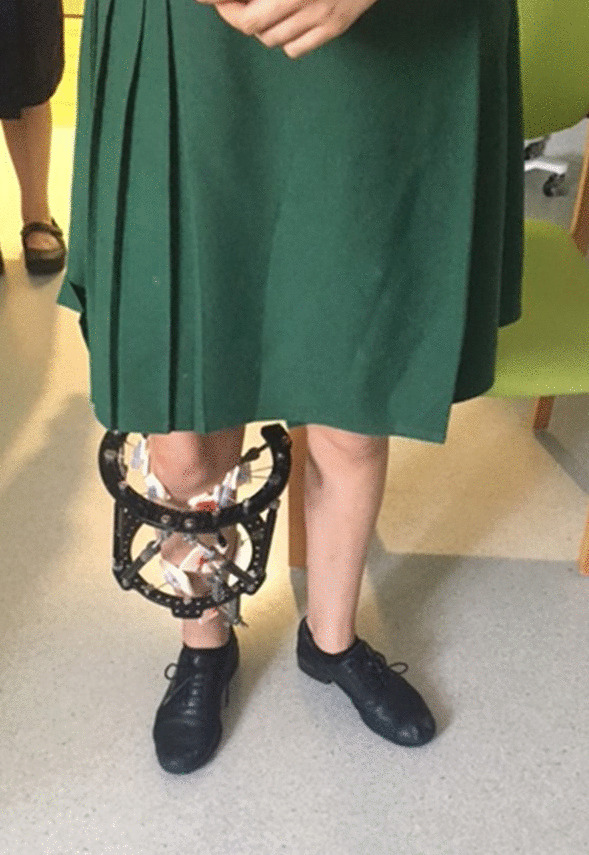
Clinical appearance following right tibial correction with a neutral foot progression angle. The left leg held with patella facing forwards to demonstrate the external foot progression prior to future correction

All osteotomies healed, and the frames were removed at a mean of 111 days (80–168) with the mean follow-up of 20 months (9–83). There was a significant improvement in the mean Kujala knee pain score, increasing from 53 (30–75) pre-operatively to 92 (54–100) at the latest follow-up (*P* < 0.001) (Fig. 1). The mean VAS significantly improved from 8 (5–9) pre-operatively to 1 (0–4) (*P* < 0.001) (Fig. 2). Thirteen patients walked with a neutral foot progression angle and patella pointing forwards (Fig. 3). One patient had a minor in-toeing of − 5°, and one had a minor out-toeing of + 10°. Both patients were content with their foot position, with no intention of further surgical intervention.

Complications included seven superficial pin site infections treated successfully with a course of oral antibiotics. One patient required intravenous antibiotics for a pin site infection. One patient required further debridement of a pin site on frame removal. One patient developed a neuroma at the site of the tibial osteotomy, which was excised with the resolution of pain.

One female patient treated with bilateral tibial osteotomies was discharged pain free at 17 months following frame removal. The patient presented 18 months later with a new onset of isolated left hip pain felt in the groin on walking. Her preoperative assessment recorded bilateral hip IR at 80° and ER at 15°, with PFA on the left measured at 50° on the CT scan and 54° on the right. The ETT was measured at 70° prior to correction. The hip pain resolved when she walked with an internal foot progression angle of − 30°. A femoral de-rotation osteotomy was performed using an intra-medullary locked nail with no complications. The patient currently walks with a neutral foot progression angle, and the hip pain has resolved.

## Discussion

Miserable malalignment syndrome is an accepted uncommon cause of anterior knee pain. It consists of proximal femoral anteversion, leading to internal rotation of the thigh, inward squinting of the patella, and significant external tibial torsion. Surgical management in patients with debilitating pain involves de-rotation of lower limb osteotomies. This study has demonstrated excellent results following proximal infra-tubercle tibial osteotomy correction using a hexapod external fixator. All patients have a significant reduction in knee pain, and the majority walk with a neutral foot progression. Only one patient has required subsequent femoral de-rotation osteotomy.

The philosophy guiding our decision-making was centred on the cause of the knee pain. With significant external tibial torsion, the ankle will be pointing outwards which may prevent roll-over during the 2nd rocker of stance. This would make walking difficult and so they place their ankle forwards by internally rotating the hip with the femoral anteversion. The inward rotation of the femur will result in an increase in tension of the quadriceps tendon with a resultant increase in patellofemoral contact pressures [11]. Lee et al. demonstrated that there was only a slight increase in pressure with 20° of either internal or external rotation but a significant increase with 30° rotation. Our results confirmed a mean foot progression angle when the patients walked with the patella pointing forwards at + 37°. This suggests that the thigh segment is internally rotated by more than 30°, as the patients normally walked with a neutral foot progression and inwardly squinting patella, which would lead to knee pain due to significant patellofemoral overload. It should also be noted that when the patients walked with the patella pointing forwards, they reported decreased knee pain.

Another knee pathology has also been shown to be associated with ETT [9, 10]. Turner and Smillie measured tibial torsion in more than 1200 consecutive patients [9]. They confirmed that patients were more likely to have unstable patellofemoral joint or Osgood–Schlatter disease with ETT greater than 25°. Other causes of ETT-related pain included fat pad impingement and chondromalacia patellae. The mean ETT seen in our study was 50°, with the largest measured at 70°. Patella instability, however, was not a presenting complaint and was also not seen clinically in our study group. We, therefore, believe that the knee pain is due to patellofemoral overload from the combination of the FPA and ETT.

Furthermore, the mean TT-TG recorded in our study group was 10.8 mm, with only one patient having a distance of 21 mm above the pathological threshold. This suggests that patella instability was not of concern in this group of patients and was not the cause of pain. The TT-TG measurement is a reliable and accurate indicator of patellofemoral instability [23].

It remains a debate as to the optimum treatment strategy for MMS. Staheli advocated femoral de-rotation osteotomy where hip IR > 85°, ER < 10°, or PFA was > 50°. A tibial correction was advised if greater than 30° ETT. Our study group recorded a maximum hip IR of 80° and a mean hip ER of 28°, with at least 10° recorded as the lowest amount. The mean PFA was 32°, with the maximum listed as 54°. As recommended by Staheli’s parameters, femoral osteotomy was only present in a few of our patients within the study group. In contrast, with a mean ETT of 50°, there was a stronger argument for tibial correction.

The optimum level of tibial corrective osteotomy requires further discussion. Proximal tibial osteotomy proximal or at the level of the tibial tubercle has been reported [2, 18–20]. Both Drexler et al. and Cooke et al. included tibial osteotomy with a realignment of the extensor mechanism with a Maquet osteotomy or tibial tubercle transfer and lateral retinacular release in cohorts of 12 patients, respectively [2, 18]. Their patients all had chronic knee pain with accompanying patellofemoral subluxation, with satisfactory results demonstrating a reduction in knee pain and symptoms reported. Cameron and Saha described 17 patients, with five reporting recurrent patella dislocation also treated with a Maquet-type osteotomy and the remaining with supra-tubercle osteotomy alone [19]. Fouilleron et al., in a larger study of 36 osteotomies in 29 patients, only five patients had patellofemoral instability. The osteotomy for all was performed at the level of the tibial tubercle. They did report one case of peroneal nerve palsy following the acute correction of ETT [20]. Our study group did not present with symptoms of patellofemoral instability, and only one case had a TT-TG greater than 20 mm (measured at 21 mm), confirming that tibial tubercle transfer was not indicated. We, therefore, decided upon an infra-tubercle osteotomy level. The use of a hexapod external fixator enabled an accurate deformity correction, a rate chosen to reduce the risk of a traction injury to the peroneal nerve and with the ability to achieve an optimum individual alignment of the tibia under patient control.

Some authors have reported a combination of both femoral and tibial osteotomy [4–6]. Bruce and Stevens undertook femoral osteotomy to correct the PFA in 14 patients [5]. All patients reported full satisfaction following surgery. In the majority, a distal tibial supramalleolar osteotomy was performed with plate fixation; the remaining seven cases were fixed with an intramedullary nail. The femoral osteotomies were also stabilised with a number of methods, including plates or nails. Significant complications included loss of fixation of one of the tibial plates and one fibular non-union. They also described one patient in their discussion, excluded from their study group due to inadequate follow-up. They underwent staged bilateral osteotomies femoral followed by tibial fixed with intramedullary nails. The patient developed bilateral temporary peroneal nerve palsies. So, they suggested performing simultaneous osteotomies of firstly the femur and then the tibia in an attempt to reduce the risk of traction injury to the nerve. Delgado et al. suggested that double-level osteotomy of the femur and tibia would predictably resolve patellofemoral pain in MMS [4]. Their study group, however, consisted of only nine patients with 13 segments treated, in whom six patients were treated by tibial osteotomy alone. They offered no instruction on decision-making as to why one patient was treated with double osteotomy, whilst others with tibia alone. They also reported several techniques in performing the tibial osteotomy, including five proximal to the tibial tubercle fixed with staples, three distal to the tubercle fixed with K-wires (one case), and two by external fixator. The remaining five by distal tibial osteotomy fixed by crossing K-wires. Leonardi et al., in a small study of three patients, stated that internal tibial rotation alone was insufficient in cases of significant deformity, as there was not enough passive hip ER [6]. This statement seems logical; the patients in our study group could walk with the patella pointing forwards, uncovering the significant ETT with a positive foot progression during gait. The mean ER of the hip was recorded at 28°, with all patients with at least 10°. Simultaneous femoral de-rotation osteotomy may be indicated in patients with no hip ER recorded at the examination, which was not seen in our cohort.

We accept that there were limitations in this study, including the retrospective analysis of a small cohort with limited follow-up. The long-term effect of increased femoral anteversion on the hip joint has been controversial. A recent meta-analysis reported that there may be an increased chance of developing osteoarthritis with increased femoral anteversion > 24° with or without hip dysplasia [24]. Therefore, the option of femoral de-rotation has been always discussed and offered to patients during counselling.

Once the patients have clinical and radiological confirmation of a healed osteotomy, with a return to full activities, they were discharged from the clinic. They were encouraged to be re-referred with new symptoms, as seen with one of our patients who subsequently underwent femoral de-rotation osteotomy. No other patients have yet to represent, but we cannot guarantee that they remain symptom free as they have not been contacted as part of the study.

Our study did demonstrate full resolution of knee pain associated with MMS following an infra-tubercle proximal tibial osteotomy and correction using a hexapod external fixator. Although acute de-rotation is a valid option, it carries the risk of common peroneal nerve stretch, despite the low possibility, it could be a catastrophic complication. The external fixator, in addition to the surgeon’s preference, would eliminate such complications and avoid the need for extra procedure (prophylactic common personal nerve release), ensures accurate correction, the ability to ensure correct alignment, and enables weight-bearing for the patient to appreciate the new position of the foot progression and adjust as necessary. Finally, the patient was encouraged to fully weight bear, maintaining the patella facing forwards. Once the foot progression was optimal, the frame was maintained till full bone healing. If the femur continues to adopt internal rotation due to the PFA, with a squinting patella and internal foot progression, the frame tends to strike the opposite leg during the swing phase of gait. This may be avoided by walking with the patella forwards.

Furthermore, the time in the frame may offer an opportunity for the patient to adapt to walking with the patella pointing forwards, as most will have walked with the patella squinting inwards for many years. We accept the difficulties associated with using an external fixator, such as pin site infection and soft tissue tethering, and the social and psychological implications of having a frame on a limb for several months. However, on balance, we believe that using a hexapod fixator with gradual correction of severe ETT in MMS with an infra-tubercle osteotomy is the optimum method to manage this complex condition.

## Conclusion

Gradual correction of severe external tibia torsion with a hexapod external fixator and an infra-tubercle osteotomy could provide an optimum method to eliminate knee pain and improve limb alignment in miserable malalignment syndrome.

## Data Availability

All data generated or analysed during this study were included in this published article.
